# Double chain system for online and offline medical data sharing ***via*** private and consortium blockchain: A system design study

**DOI:** 10.3389/fpubh.2022.1012202

**Published:** 2022-10-11

**Authors:** Chaoran Li, Jusheng Liu, Guanyu Qian, Ziyi Wang, Jingti Han

**Affiliations:** ^1^School of Economics and Management, Shanghai University of Sport, Shanghai, China; ^2^School of Economics and Management, Shanghai University of Political Science and Law, Shanghai, China; ^3^Business School, Hunan University, Changsha, China; ^4^School of Humanities, Shanghai University of Finance and Economics, Shanghai, China; ^5^School of Information Management and Engineering, Shanghai University of Finance and Economics, Shanghai, China

**Keywords:** blockchain, Internet medicine, electronic health records, data sharing, data management

## Abstract

With the informatization development and digital construction in the healthcare industry, electronic medical records and Internet medicine facilitate people's medical treatment. However, the current data storage method has the risk of data loss, leakage, and tampering, and can't support extensive and secure sharing of medical data. To realize effective and secure medical data storage and sharing among offline medical institutions and Internet medicine platforms, this study used a combined private blockchain and consortium blockchain to design a medical blockchain double-chain system (MBDS). This system can store encrypted medical data in distributed storage mode and systematically integrate the medical data of patients in offline medical institutions and Internet medicine platforms, to achieve equality, credibility, and data sharing among participating nodes. The MBDS system constructed in this study incorporated Internet medicine care services into the current healthcare system and provided new solutions and practical guidance for the future development of collaborative medical care. This study helped to solve the problems of medical data interconnection and resource sharing, improve the efficiency and effect of disease diagnosis, alleviate the contradiction between doctors and patients, and facilitate personal health management. This study has substantial theoretical and practical implications for the research and application of medical data storage and sharing.

## Introduction

With the informatization development and digital construction in the healthcare industry, the growth of medical data is showing a blowout trend. At present, many hospitals store the patients' electronic health records (EHRs) in the database to record the entire treatment process. When a doctor needs a patient's treatment and prescription records, the service system will extract the required data from the database for the doctor (1). Medical records are essential for the diagnosis and follow-up of patients. For the patient, diseases can lead to complications. In this case, the accuracy of the diagnosis is affected by the volume and accuracy of the historical health and medical information of the patient obtained by the doctor (2). By sharing the patient's health and medical data, doctors can access the patient's EHR and understand the patient's medical history and the corresponding treatment plan, to diagnose and treat the patient more accurately and comprehensively. There are various medical data sources, including clinical medical records, outpatient visits, relevant medical examination results, etc. (3). But, most hospitals don't establish cross-institution medical data sharing channels. Doctors in different hospitals are not able to access the patients' EHRs in other hospitals, resulting in information silos. Therefore, it is necessary to share patients' EHRs across hospitals in time to facilitate disease diagnosis and treatment (4). Besides, with the increasing demand for personal health management, medical institutions hope to collect health and medical information through the exchange and sharing of medical data (5). A large amount of medical data can support the development and application of recommendation systems in the medical industry (6, 7). Furthermore, the current data storage method has the risk of data loss, and tampering, and can't support extensive and secure sharing of medical data. Electronic medical data, such as prescription records, and medical records, may be tampered with or missing information, which leads to data lack of credibility (8, 9). In particular, the authenticity and fairness of medical information provided in the event of medical disputes cannot be guaranteed (10). Therefore, it is necessary to use the new electronic data storage technology to store, share and notarize data, and provide legal evidence for medical disputes and medical negligence while sharing medical data among doctors, hospitals, and other relevant stakeholders (11).

Blockchain technology, the underlying technology of Bitcoin, is characterized by transparency, anonymity, autonomy, and tamper-proof (12–14). As a technology, it is widely used in voting, supply chain, healthcare, the Internet of Things, finance, and other fields (15). In the healthcare industry, blockchain technology, as a new type of electronic data storage technology, can help hospitals, doctors, patients, and scientific researchers share and access medical data quickly and safely (16). Currently, the application and research of blockchain in the field of healthcare have received wide attention (17–20). Therefore, this research used blockchain technology to design a medical data sharing and application model between medical institutions to help solve real-life problems like medical data storage concentration, security sharing difficulties, privacy leaks, excessive dependence on authority institutions for data credibility, etc. This model helps to realize the sharing and application of decentralized, safe, efficient, and tamper-proof health and medical information, and to construct an equal and credible medical ecological environment.

The wide application of Internet technology has brought about great changes in the way people obtain disease knowledge and medical information. Online services, such as online medical consultation services, and disease information retrieval provided by Internet medical care platforms, can meet the growing health needs of the public (21, 22). Internet medical care platforms, such as online health community platforms, can solve the shortage of medical resources and uneven distribution of medical resources to a certain extent, thereby alleviating doctor-patient conflict and improving the doctor-patient relationship (23). The development of Internet medical care has generated massive online health and medical data (such as medical knowledge, online medical records, public communication records, etc.). Besides the health records from the hospitals, if a doctor can obtain a patient's health data from the online healthcare platforms, it will help the doctor to diagnose the diseases, as well as the later health management of patients. Indeed, the massive online information has high-quality and low-quality information, the low-quality information on the platform will mislead patients and affect their disease treatment. In this study, For ease of expression and understanding, we define as below: Health and medical information (HMI) refer to all information or data including health information/data and medical information/data about people's physical health and disease treatment (5, 24). Therefore, in the general application environment of Internet medicine, in addition to the use of blockchain technology to realize the storage and sharing of HMI between traditional offline hospitals, it is necessary to integrate the HMI of the traditional offline hospitals and the HMI of the Internet medicine platforms. The digital signature, endorsement, and traceability technology of blockchain can effectively avoid the generation and dissemination of low-quality information on Internet medicine platforms. In this way, we can realize the sharing and use of HMI between different offline and online medical channels. Internet medicine platforms, through the seamless connection with offline hospitals, can give full play to the role of online medical services in disease prediction, diagnosis, and follow-up. At the same time, we can integrate online medical services into the current health care system to improve the utilization efficiency of medical resources. However, there is a lot of information on the Internet medicine platform, and the low-quality information on the platform will mislead patients and affect their disease treatment.

In summary, this research explores how to use blockchain and Internet medicine technology to design a medical blockchain system to build an equal and credible medical ecological environment, and realize online and offline hybrid medical applications to solve the problem of medical information interconnection and resource sharing. In this paper, we define as below: offline medical treatment refers to patients going to traditional physical hospitals to seek doctors for disease diagnosis and treatment, and patients and doctors can communicate face-to-face. Online medical treatment refers to patients seeking doctors for disease diagnosis and treatment on the Internet medical platforms. Patients and doctors can only communicate through the Internet. Technically, this study systematically integrates the HMI of offline medical institutions (such as community hospitals, specialized hospitals, general hospitals, rehabilitation and healthcare hospitals, nursing homes, health examination centers, and scientific research institutions) and online Internet medical platforms by constructing a medical blockchain system. With the help of decentralization, anti-tampering, traceability, and joint participation of the blockchain, we can achieve equality, credibility, and data sharing among participating nodes in the medical ecosystem. To improve data security, reduce the computational pressure and avoid too many nodes on a blockchain, this study combined the consortium blockchain and private blockchain to build an integrated medical blockchain double chain system (MBDS) for the storage and sharing of HMI in different offline medical institutions and Internet medicine platforms. Given all this, the MBDS system can store encrypted medical data in distributed storage mode and systematically integrate the patients' medical data from different sources, to achieve equality, credibility, and data sharing among participating nodes. The MBDS system constructed in this study incorporated Internet medicine care services into the current healthcare system and provided new solutions and practical guidance for the future development of collaborative medical care. This study helped to solve the problems of medical data interconnection and resource sharing, improve the efficiency and effect of disease diagnosis, alleviate the contradiction between doctors and patients, and facilitate personal health management. This study has substantial theoretical and practical implications for the research and application of medical data storage and sharing.

The difference between this study and previous studies is as below: firstly, it used blockchain technology to integrate HMI of the online and offline channels, which contributed to building an equal and credible medical ecological environment and realizing the online and offline hybrid medical application. Secondly, the private blockchain and consortium blockchain are combined with different consensus mechanism algorithms to build a medical blockchain dual-chain system to improve the operating efficiency of the blockchain system and reduce energy consumption. Thirdly, by combining the on-chain storage of the blockchain with cloud storage, the cloud storage server is responsible for storing the encrypted original medical data, and the blocks on the blockchain are responsible for storing the hash value and data address of the encrypted data on the cloud server and information summary, the method can realize the safe and efficient storage, privacy protection and anti-tampering of the HMI with large storage capacity.

The structure of this paper is as follows: Section 2 introduces the related literature and the technology used in this study; section 3 describes our research objectives; section 4 illustrates the framework and design details of the MBDS system; section 5 illustrates the use process of the MBDS system and how to achieve online and offline hybrid medical treatment; section 6 summarizes this research and discusses the deficiencies and improvement directions of this research.

## Related work

### Literature review

In the healthcare industry, medical data is scattered in various medical institutions, there are no unified data storage and sharing standards in the different medical institutions, and permissions control of medical data access is backward. All these have brought great difficulties to the exchange and sharing of medical data, seriously affecting the validity and value mining of the medical data (5). How to realize the safe sharing of medical data is the focus and hot spot in the current field of medical data application. The realization of this goal mainly includes two aspects: (a) Authorized distribution and secure transmission of data; (b) Privacy protection of personal data (25). Blockchain technology provides an effective solution to the problem of the safe sharing of medical data. The application and research of blockchain in the field of healthcare have received extensive attention and great attention. Healthbank, a global innovator in digital health, is actively exploring the application of smart contracts in blockchain (17). Gem Health cooperated with the Philips Blockchain Lab to use blockchain technology to create a medical ecosystem connected to a common data infrastructure, and solve the balance between patient-centric care and operational efficiency (18). As a whole, blockchain technology can be used to store and share medical data among medical industry participants and stakeholders (such as hospitals, patients, insurance companies, etc.), which enables data users to share and apply health and medical information securely and efficiently (8, 26).

At present, the study of blockchain in the field of healthcare includes medical data protection, medical data storage and sharing, and medical data application (20, 24, 27). Mettler (28) describes the possible impact goals and potential of blockchain technology in the field of healthcare. In terms of medical data protection, Dagher et al. (24) proposed a blockchain-based framework, Ancile, which is based on the smart contract of Ethereum blockchain to strengthen access control and avoid data confusion. While protecting patients' sensitive information and privacy, medical data can be accessed safely, conveniently, and efficiently by patients, data providers, and third parties. Albalwy et al. (29) described ConsentChain, as a block-based system, which can allow patients to grant or withdraw medical data access. In terms of data storage and sharing, Azaria et al. (27) built a medical information sharing platform (MedRec) based on the Ethereum blockchain, which combined medical blockchain and big data. Liu et al. (15) proposed a blockchain-based privacy-preserving electronic medical record data sharing system, BPDS, which stores the original medical record information in a cloud storage server. Zhang et al. (8) designed a consortium medical blockchain system based on the PBFT algorithm to store and share medical data. In addition, some data-sharing systems based on blockchain technology are constantly being proposed and studied (13, 30). Regarding medical data application, Miyachi and Mackey (20) proposed a modular hybrid privacy-preserving framework leveraging off-chain and on-chain blockchain system design applied to three different reference models that illustrate how blockchain can enhance healthcare information management. Yue et al. (31) proposed a blockchain-based App (Healthcare Data Gateway). The system not only enables patients to easily and securely own, control, and share their data, but also allows untrusted third parties to access and process medical data while ensuring patients' privacy.

In summary, in the healthcare industry, blockchain technology, as a new data storage technology, can realize the sharing and access of health data while providing data notarization. By using blockchain technology, we can realize the sharing and application of decentralized, safe, efficient, and immutable health data among various institutions.

### Preliminaries

#### Blockchain

The blockchain is a distributed database that contains an ordered list of records linked together through a chain of blocks, which it is jointly participated in and maintained by multiple independent nodes (32, 33). Compared with the traditional distributed database maintained by a central server, blockchain is a decentralized distributed database in which the data stored on the blockchain is jointly maintained by all nodes in the blockchain network, each of the nodes holds a complete set of data. To ensure the safety and reliability, the consensus mechanism in the blockchain network is crucial, which determines who recording the transaction data and how to check the validity of new blocks (2, 30, 34).

Blockchain can be classified into three categories: public blockchain, consortium blockchain, and private blockchain. (a) The public blockchain refers to everyone involved who can review transactions and participate in the consensus-building process. For example, Bitcoin and Ethereum are both public blockchains. (b) Consortium blockchain, only nodes (authorized organizations) in the system can access and manage the blockchain. Usually, there is a commercial cooperation relationship between nodes. The data in the consortium blockchain can be public, private, or partially decentralized. (c) Private blockchain, nodes will be restricted. Not every node can participate in this kind of blockchain. The written permission of a blockchain is only in the hands of a certain person or organization, and there is strict permission management for data access. For example, the internal use of blockchain for document management within an enterprise is the use of private blockchain. No matter what type of blockchain it is, it has its advantages and applicable application scenarios. Sometimes we need a public blockchain because it is convenient and there are many individuals involved, but sometimes we may need a consortium blockchain or a private blockchain to control read and write permissions, depending on what kind of services we need to provide and what kind of scenario we are applying to Niranjanamurthy et al. (35).

Combined with the research work in this paper, this study will introduce two types of blockchains, private blockchain, and consortium blockchain, to store and manage the patient's HMI, which will help solve the problems of medical information interconnection and resource sharing, also contribute to improving the doctors' disease diagnosis efficiency and treatment effect for patients. In the medical blockchain system constructed in this study, each hospital has a private blockchain to store the HMI of its patients. The hospitals, health authorities, third-party regulatory agencies, medical insurance companies or organizations, etc. negotiate and establish consortium blockchain, which is used to realize cross-organization HMI sharing.

#### Consensus mechanism

The consensus mechanism is the core technology for blockchain because it determines whether the new block is validated and who keeps the record. Therefore, it impacts the security and reliability of the whole blockchain system (2, 34). The consensus algorithm in the blockchain system is a confirmation mechanism for the blockchain system to detect the legitimacy of data and add blocks to the blockchain (8). There are many consensus algorithms in practical applications, such as Proof of Work (PoW) used in Bitcoin, Proof of Stake (PoS), Delegated Proof of Stake (DPoS) (15), Practical Byzantine Fault Tolerance (PBFT) (8, 36), as well as Proof of Information (PoI) (37).

DPoS is the consensus algorithm used by the BitShares project (15). All participating nodes in the blockchain have voting rights. One hundred and one equity representative nodes that are selected through a fair and democratic voting method are in turn responsible for creating and verifying blocks in turn. In the subsequent algorithm execution process, all nodes can re-vote freely based on the performance of the equity representatives, and dynamically manage the selected 101 equity representatives to ensure the reliability and stability of the selected equity representative nodes in the blockchain system (16). Compared with PoW and PoS, DPoS is a faster, more effective, more decentralized, more flexible, and more energy-saving consensus mechanism. In addition, this algorithm can effectively reduce the number of participating accounting nodes and achieve rapid consensus verification (34). PBFT is a consensus algorithm introduced in 1999 by Barbara Liskov and Miguel Castro (36). PBFT was designed to work efficiently in asynchronous (no upper bound on when the response to the request will be received) systems. It is optimized for low overhead time. PBFT tries to provide a practical Byzantine state machine replication that can work even when malicious nodes are operating in the system. Nodes in a PBFT-enabled distributed system are sequentially ordered with one node being the primary (or the leader node) and others referred to as secondary (or the backup nodes). It is noted that any eligible node in the system can become the primary by transitioning from secondary to primary (typically, in the case of a primary node failure). The goal is that all honest nodes help in reaching a consensus regarding the state of the system using the majority rule. A practical Byzantine Fault Tolerant system can function on the condition that the maximum number of malicious nodes must not be ≥1-third of all the nodes in the system. As the number of nodes increases, the system becomes more secure (8).

In this study, we constructed a medical blockchain double-chain system by simultaneously using both consortium blockchain and private blockchain. DPoS is used as the consensus algorithm in the consortium blockchain established by hospitals, health authorities, third-party regulatory organizations, insurance companies, and other relevant institutions or organizations. DPoS algorithm has the advantages of lower energy consumption, more decentralization, and faster confirmation speed. For a consortium blockchain in this study, the participating nodes are relatively stable and the change is less, thus, DPoS can give full play to the role of each equity representative node, and realize fast transactions in the face of a large number of transactions. Furthermore, this research improves the election method of equity representative nodes in the DPoS algorithm by considering nodes' offline business performance and credit, which can better urge each node to play the role of miners and validators.

Regarding the private blockchain established within each hospital mainly for doctors and patients to store the generated HMI, we use PBFT as the consensus algorithm. Because different hospitals have huge differences in the number of doctors and the number of patients are constantly changing, the DPoS algorithm requires that the number of participating nodes in the blockchain system is always maintained at more than 101, so the DPoS algorithm cannot meet the requirements of the hospital's internal private blockchain. This paper studied the use of the PBFT algorithm as the consensus algorithm of the hospital private chain. The minimum number of participating nodes required by this algorithm is 4, which can well-meet the requirements of large and flexible participation nodes in the private blockchain of each hospital.

#### Proxy re-encryption

Proxy re-encryption (PRE) is a conversion mechanism that can be used between ciphers. It was originally proposed by Balze et al. (38). This technique solves the problem of transferring encrypted records between nodes without sharing symmetric keys by using a proxy. The proxy is responsible for reconstructing an encrypted message in such a way that other users can use their private key to decrypt the received encrypted documents to obtain the plaintext, even if the documents were not originally encrypted with their associated public key (39). This system allows secure sharing between parties without fully decrypting the document during the transfer process (24). The purpose of using proxy re-encryption is to solve the inconvenience of sharing data, reduce the burden on users, and enhance data reliability and security. In the process of proxy re-encrypting, each participator cannot individually retrieve any plaintext messages alone.

The specific working process involves three roles: data owner, data user, and proxy. We take an example to introduce how it works. When the data owner (Alice) wants to share the encrypted document with the data user (Bob), Alice generates a proxy re-encryption key for Bob and transmits the proxy re-encryption key to a third-party semi-trusted proxy through a secure channel. The semi-trusted proxy uses the proxy re-encryption key to re-encrypt the encrypted document according to the proxy re-encryption algorithm and send the re-encrypted document to Bob. After Bob obtains the re-encrypted document, he can use his private key to decrypt the re-encrypted document and obtain the plaintext after decryption. The specific workflow is as follows:

(1) KeyGen: generating public and private keys. Alice and Bob request the Key Management Center (KMC) to generate their public and private key pairs, and KMC generates Alice and Bob's public and private key pairs (*PK*_*A*_,*SK*_*A*_) and (*PK*_*B*_,*SK*_*B*_), and return them to Alice and Bob, respectively. *PK* is the public key, and *SK* is the private key.
(2) Encryption (Enc): Encrypting plaintext. Alice encrypts the plaintext *M* with her public key *PK*_*A*_, *C*_*P*_*K*__*A*__ = *Enc*(*PK*_*A*_, *M*), where *M* is the document that Alice wants to transmit to Bob.
(3) Transfer (Trans): Transferring encrypted document. Alice sends the encrypted document *C*_*P*_*K*__*A*__to the semi-trusted proxy. At the same time, Alice uses Bob's public key *PK*_*B*_ and her private key *SK*_*A*_ to generate a re-encryption key *RK*_*A*→*B*_, and sends the re-encryption *RK*_*A*→*B*_ to the semi-trusted proxy.
(4) Re-Ecrpytion (ReEnc): Proxy re-encrypting the encrypted document. Using the re-encryption algorithm *ReEnc*(*C*_*P*_*K*__*A*__, *RK*_*A*→*B*_): given the encrypted document *C*_*P*_*K*__*A*__ corresponding to the public key *PK*_*A*_, the proxy uses the re-encryption key *RK*_*A*→*B*_ provided by Alice to convert the encrypted document *C*_*P*_*K*__*A*__ into the re-encrypted document *C*_*P*_*K*__*B*__ by using this re-encryption algorithm.
(5) Trans: Transferring the re-encrypted document. The proxy sends the re-encrypted document *C*_*P*_*K*__*B*__ to Bob.
(6) Decryption (Dec): Decrypting the re-encrypted document. Bob can use the private key *SK*_*B*_ corresponding to the public key *PK*_*B*_to decrypt the re-encrypted document *C*_*P*_*K*__*B*__, *M* = *Dec*(*SK*_*B*_, *C*_*P*_*K*__*B*__), then to obtain the original plaintext *M*.

The above process reduces the workload and resource consumption of Alice as the data owner. Alice only needs to generate a re-encryption key for Bob, and the transmission, transformation, and storage of the document are all processed by the semi-trusted proxy. The proxy re-encryption mechanism provides convenience for users to share data, which can not only reduce the burden of users but also enhance the reliability and security of data (40). The process and advantages are conducive to the storage and transmission of HMI of medical institutions and patients, then to achieving secure and efficient transmission of medical data.

#### Public encryption with key search

Public encryption with key search (PEKS) was proposed by Boneh et al. (41), which aims to search over the encrypted data in asymmetric settings. Usually, a keyword *w* is extracted from a message *M*. While storing the data, the keyword will be encrypted with the user's public key and stored in the server. By using the private key corresponding to the user's public key to generate a trapdoor, the visitor can search for the encrypted keyword *C*_*w*_. The PEKS algorithm mainly includes the following four parts:

(1) KeyGen(λ): Key generation algorithm. The user *i* inputs a security parameter λ to obtain the public and private key pair (*PK*_*i*_,*SK*_*i*_), where *PK*_*i*_ is the public key and *SK*_*i*_ is the private key.
(2) Enc(*PK*_*i*_,*w*): Keyword encryption algorithm. Given the keyword *w* of a document, the user *i* encrypts the keyword with his public key *PK*_*i*_, and outputs the encrypted keyword *C*_*w*_.
(3) Trapdoor(*SK*_*i*_,*w*′): Trapdoor generation algorithm. Input the private key of the user *i* and the target keyword *w*′, and output the trapdoor $T_{w^{{}^{\prime }}}$corresponding to the target keyword *w*′.
(4) Test($T_{w^{{}^{\prime }}}$,*C*_*w*_): Test algorithm. Input the trapdoor $T_{w^{{}^{\prime }}}$ and the ciphertext *C*_*w*_, when the trapdoor and the ciphertext correspond to the same keyword, that is, $T_{w^{{}^{\prime }}}=C_{w}$, output “True,” otherwise output “False”.

The MBDS system constructed in this study will encrypt the keywords extracted from the patient's HMI, then it will store the encrypted keywords, the patient's public key, and the encrypted patient's HMI in the blockchain system. When a user needs a patient's medical data for a certain disease, a proxy can retrieve the record corresponding to the keyword through the PEKS algorithm and obtain the storage addresses. Then, by using PRE technology, the patient instructs the proxy and delegates the proxy to transmit the encrypted HMI of the corresponding storage addresses to the data users who need to access the patient's HMI. The PEKS algorithm retrieves the encrypted ciphertext of the keywords stored on the blockchain, which can realize the data retrieval function under the premise of protecting the privacy of the patient, avoiding the transmission and decryption of a large amount of useless information, and realizing the improvement of the efficiency of data retrieval and reading and saving energy.

## Research objective

As we know, it is necessary to ensure that the benefits of the designed blockchain-based information management system features can be applied to a variety of health-related data, while also maintaining the privacy and security of healthcare data governance. These design objectives are very necessary and crucial in breaking down data silos to ensure the maximal utility of healthcare data for all relevant stakeholders in the medical industry (20, 42). Therefore, in this study, we aimed to conceptualize, design, and implement a blockchain-based information management system framework to store and manage HMI. The use of blockchain technology can safely and transparently store and manage HMI. The MBDS system constructed in this study aimed to realize the shared application of multi-source HMI between offline medical institutions and Internet medicine platforms. This study adopted the idea of a hybrid chain to combine the private blockchain for each hospital and the consortium blockchain between different related organizations in the medical industry, and used proxy re-encryption (PRE) and public encryption with keyword search (PEKS) technology to construct a medical blockchain double-chain system (MBDS). In this system, private blockchain and consortium blockchain were used to store and manage the patient's HMI, which helps improve the diagnosis. Each hospital operates a private blockchain that stores the patient's HMI in the hospital. The hospitals and related stakeholders, such as health authorities, research institutions, online health community platforms, and insurance companies, negotiated to manage a consortium blockchain, which keeps records of the secure indexes for the healthcare data.

Thus, referring to the existing research (2, 20, 43), the constructed MBDS system needs to achieve the following design objectives:

(1) Data security. HMI involves the patients' privacy, and the disclosure of information without the patient's consent will hurt the patients' lives. In addition, HMI concerns the health of the users, and an error and omission of information may affect the diagnosis and treatment of the patient's disease. Therefore, the designed MBDS system must ensure the security of stored data, including data confidentiality and integrity. Firstly, the HMI is encrypted and signed by asymmetric encryption technology to prevent data errors and omissions to happen; secondly, HMI is hashed and stored in the next block to prevent data errors through a hash algorithm; thirdly, the user's public key is used to replace the user's real identity; lastly, data auditing and access control are used to monitor all data access activities to ensure the confidentiality and integrity of data. Moreover, as an independent third-party organization, the nodes of health authorities in each blockchain need to play a supervisory role to ensure that the data on the chain will not be tampered with, and at the same time serve as a participating node to store all the data on the blockchain to avoid data loss. In addition, the MBDS system uses a combination of the hospital's internal private blockchain and the consortium blockchain among hospitals to improve data security. The above actions help to achieve data security storage.
(2) Data sharing and access control. The traditional way of healthcare information storage makes it difficult to integrate and connect the data of different medical institutions, which hinders the sharing of medical data. The MBDS system can integrate the HMI into the Internet medicine platforms and offline medical institutions to realize the cross-institution and cross-channel sharing of the patients' HMI. The doctors can obtain the most comprehensive medical information about patients. Thereby, the MBDS can improve the efficiency and effectiveness of the patient's disease diagnosis. As the owner of the HMI, the patients need to have full control over the data. The patients can determine whether other people can access their own HMI based on their situation and the application of others. The person with authorization can access the patient's data stored in the MBDS system. In addition, the patients can set the validity period for others' access authority and revoke others' access authority at any time.
(3) Privacy-preservation. While storing data securely, the data storage must be anonymized. All user identity information is stored using the user's identity code, that is, the user's public key, to avoid any information about the user's real identity in the stored data. Furthermore, the HMI and its extracted keyword stored in the block and cloud storage server should be encrypted, so no readable information can be obtained in the undecrypted state.
(4) Secure search. The MBDS system only allows doctors or institutions authorized by the patient to query the patient's HMI, and unauthorized units or individuals cannot access the data. The system uses PEKS to encrypt the keyword data stored in the blockchain. In the process of accessing the data, the visitor needs to use PEKS to retrieve the data that matches the search keyword trapdoor. This method can prevent other nodes in the distributed database from knowing the medical data keywords related to the patient's public key, which better protects the patient's privacy, and at the same time reduces the burden caused by unnecessary data access.
(5) The operability and scalability of the system. Convenient operability is a primary and important requirement of the medical blockchain, which must consider the convenient use of patients with different backgrounds. Regarding scalability, the system needs to have the ability to provide function extensions according to the requirements of participating institutions. It can not only store medical data of medical institutions but also store data of related institutions or enterprises in the medical industry. For example, to support the business and work of medical insurance companies and scientific research institutions, it is particularly necessary to integrate the user's healthcare and exercise information collected from mobile health devices. Comprehensive and all-sided medical data can be widely used in the health and medical industry to solve the problems of medical service quality supervision and control, as well as the timeliness and accuracy of medical insurance claims.
(6) Support the construction of an equal and credible medical ecological environment. All participating nodes in the system are equal and reliable. Based on ensuring the operability and scalability of the system, with the large-scale practical application of the system, all participating institutions in the medical industry are equal and credible, thus, it contributes to the formation of an equal and credible medical ecological environment in the society.

## MBDS system design

### System architecture

This study combined private blockchain and consortium blockchain to design a medical blockchain double-chain system (MBDS). By applying this system to the medical industry for large-scale use, an equal and credible medical ecological environment can be built. The MBDS system systematically integrates the medical resources and online doctor-patient information from offline medical institutions and Internet medicine platforms and integrates Internet medical care into the current healthcare system, which aims to realize the sharing and use of medical information between offline medical institutions and Internet medicine platforms. The constructed system includes plenty of private blockchains used in each hospital and a consortium blockchain between various institutions such as hospitals and insurance companies, to improve data security on the blockchain, reduce computational pressure and avoid the situation of too many nodes on a blockchain. Thereinto, the private blockchains store medical data generated when patients visit hospitals, and each hospital builds its private blockchain system. The only one consortium blockchain stores the transaction data between the institutions and the information summary of the HMI stored in the private blockchains (such as patient ID, encrypted patient's HMI, HMI hash value, HMI belonged blockchain ID, block ID, transaction order ID, etc.), which is used for cross-hospital data retrieval. This double-chain system can achieve lower computational pressure, faster transactions, lower operating costs, and better privacy protection.

**Figure 1:**
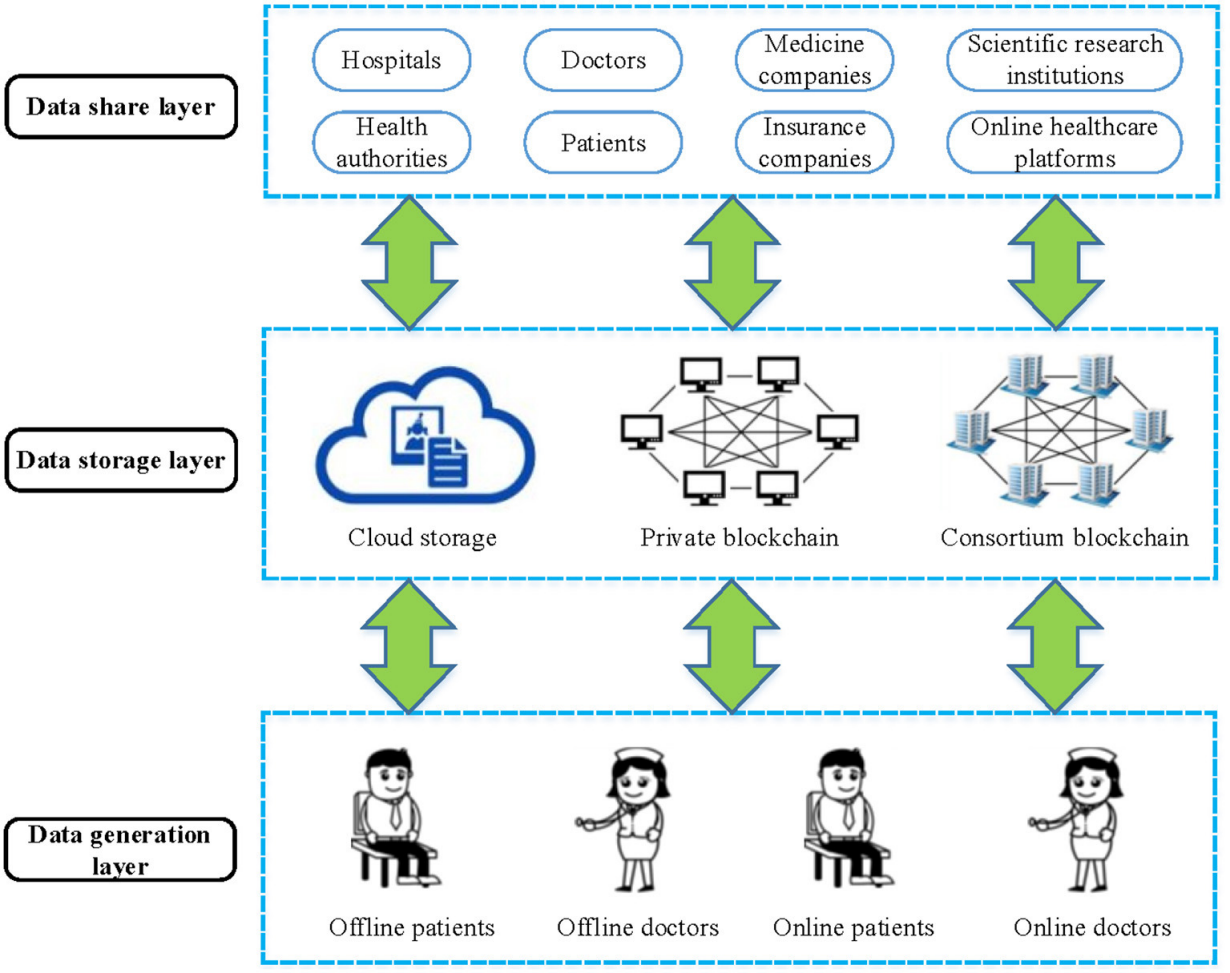
The architecture of the MBDS system.

The framework of the MBDS system is shown in Figure 1. The system can be divided into three layers, namely the data generation layer, data storage layer, and data sharing layer. The functions of each layer are as follows:

(1) Data generation layer. In this layer, the patient sees a doctor in an offline or online hospital, and the patient's HMI is generated by the offline doctor or online doctor. Then, the doctor encrypts the patient's HMI and sends it to the patient for verification and confirmation. As the owner of the data, the patient has complete control over the data, and can control the access rights of the data to determine who can access it, but does not have the right to write or modify the data.
(2) Data storage layer. The function of this layer is to store the HMI and address the index generated during the patient's visit. The module includes three parts:

- Cloud storage. The medical data sources are various and numerous. Considering the high cost and storage capacity limitations of storing large amounts of data directly on the blockchain, the raw medical data will be encrypted and stored in the cloud storage under the blockchain. At the same time, the data generation timestamp, transaction type, transaction content, doctor ID, patient ID, encrypted HMI keywords, encrypted HMI storage address, and HMI hash value is stored in the transaction slip in the block body on the blockchain. Access control is determined by access permissions, and different nodes have different access control permissions.
- Private blockchain for hospitals. The private blockchain is established within the hospital, the participants are doctors and patients of the hospital and health authorities. The MBDS system uses a private blockchain to store the returned information after the cloud storage server under the blockchain stores the medical data. Each time a piece of information is returned, a transaction slip is established and stored in the block body. In the MBDS system, the right to use the patient's HMI is completely controlled by the patient, who can authorize data users to access relevant data or revoke authorization on time. Patients can pre-define access permissions in the smart contracts to improve convenience while ensuring secure data sharing. In addition, each access request and access activity must be recorded in the blockchain for future audits or investigations. For offline hospitals, each hospital corresponds to a private blockchain. On the contrary, the Internet medicine platform takes the platform as the object. A platform builds a private blockchain for storing and sharing patients' online HMI.
- Consortium blockchain for institutions. The participants of consortium blockchain include hospitals, Internet medicine platforms, health authorities, insurance companies, scientific research institutions, and medicine companies. The hospitals extract information summaries (such as block ID, transaction slip ID, patient ID, encrypted HMI keyword, and HMI hash value) from the data stored on their private blockchains and store them in the consortium blockchain for other nodes to retrieve and share information.

(3) Data sharing layer. The participants in this layer include offline hospitals, Internet medicine platforms, doctors, patients, health authorities, insurance companies, scientific research institutions, medicine companies, etc. At this layer, data users (such as doctors and insurance companies) can apply for access to patients' HMI, and systematically analyze the patient's full-cycle medical data to provide patients with accurate disease diagnosis and treatment or timely insurance claims. For researchers, scientific research can be carried out based on the collected patient data. In short, authorized data users can retrieve the data stored in transaction slips on the blockchain and access data stored in a cloud storage server under the blockchain.

### Double-chain system

#### User role

The MBDS system constructed in this study consists of five roles, namely, data owner, data user, authority center, proxy, and miner. In the hospital's private blockchain, the participating nodes are mainly composed of a hospital, doctors, patients, and health authorities. In the consortium blockchain, the participating nodes are mainly composed of hospitals, Internet medicine platforms, health authorities, insurance companies, scientific research institutions, medicine companies, and other relevant organizations. Each node plays at least one role. In addition, the nodes like authority centers, proxies, and miners need to store full copies of the data on the blockchain, while other roles can store partial copies of the data based on business needs.

(1) Data owner. Data owners have their HMI and control over the data. Besides the data owners can share the data with other individuals or organizations but do not have the right to write and modify the data. A data owner can use the public key to encrypt and sign the original HMI and its keywords to store in the cloud storage server bundled with the blockchain, then he will store the hash value of stored HMI, storage address, encrypted HMI keywords, and access control policy on the blockchain for others to retrieve while preventing malicious tampering of data and protecting the privacy of patients. The data owners control the transmission and access rights of their HMI. In the process of data sharing, the data owner needs to rely on the proxy to re-encrypt the shared data and share the encrypted HMI with others.

(2) Data user. Data users can read and write shared files according to access control policies. Data users who are authorized by the data owner can obtain the re-encrypted HMI from the proxy by sending an authentication request, and then decrypting the received ciphertext with their private keys to obtain the plaintext of the HMI. Specifically, when data users need to update a file, they are the writer, and when they look up a file, they are the reader.

(3) Authority center. There are some nodes, which play an important role in the blockchain node network due to their business scale and institutional nature. The authority centers represent some of the participating nodes in the blockchain. In the MBDS system, the authority centers within the private blockchain are hospital and health authorities; the authority centers within the consortium blockchain are a certain number of representative institutions or organizations who are selected from various participating institutions, such as hospitals, Internet medicine platforms, health authorities, insurance companies, and scientific research institutions. The authority centers need to have a good infrastructure and the ability to invest resources to maintain and upgrade the hardware facilities in the blockchain system. Therefore, the authority centers need to store a full set of data copies on the blockchain.

(4) Proxy. The proxy employs PRE technology to realize data transmission and sharing for data users, and at the same time assists data users in PEKS retrieval of data on the blockchain. Due to the technical and resource advantages of the authority centers, the proxies generally come from authority centers.

(5) Miner. Miners are responsible for verifying file writing and reading transactions. It is determined by the consensus mechanism in the blockchain which node is responsible.

#### Client category of MBDS system

Different users in the MBDS system have different functional requirements for the system. In practical applications, the MBDS system provides three types of clients for different user roles: complete client, lightweight client, and online client. (a) Complete client: it stores copies of all data. Users who use this client are generally authority centers and proxies, and provide external services such as data query and download to other users through interfaces. (b) Lightweight client: it does not save all data, but only saves data blocks related to itself. When it needs to access other data or data verification, it needs to query other nodes or authority centers. (c) Online client: it browses in web mode, and provides users with simple operation and access services without saving data copies. Five types of users can choose to use different clients or a combination of them according to their own needs. For example, patients, as data owners, will use lightweight clients to save their data locally, which makes it faster and more convenient to load data when accessing them. At the same time, they will also use online clients to view HMI anytime, anywhere. As an authoritative center and data user, the hospital will choose to use a complete client to save a copy of all the data. After obtaining the authorization of the data owner, the hospital can quickly access relevant documents and provide data query and verification services for other nodes. Health authorities, as the authority centers, will also use complete clients to save a full set of data copies of the blockchain.

#### Node network of MBDS system

**Figure 2:**
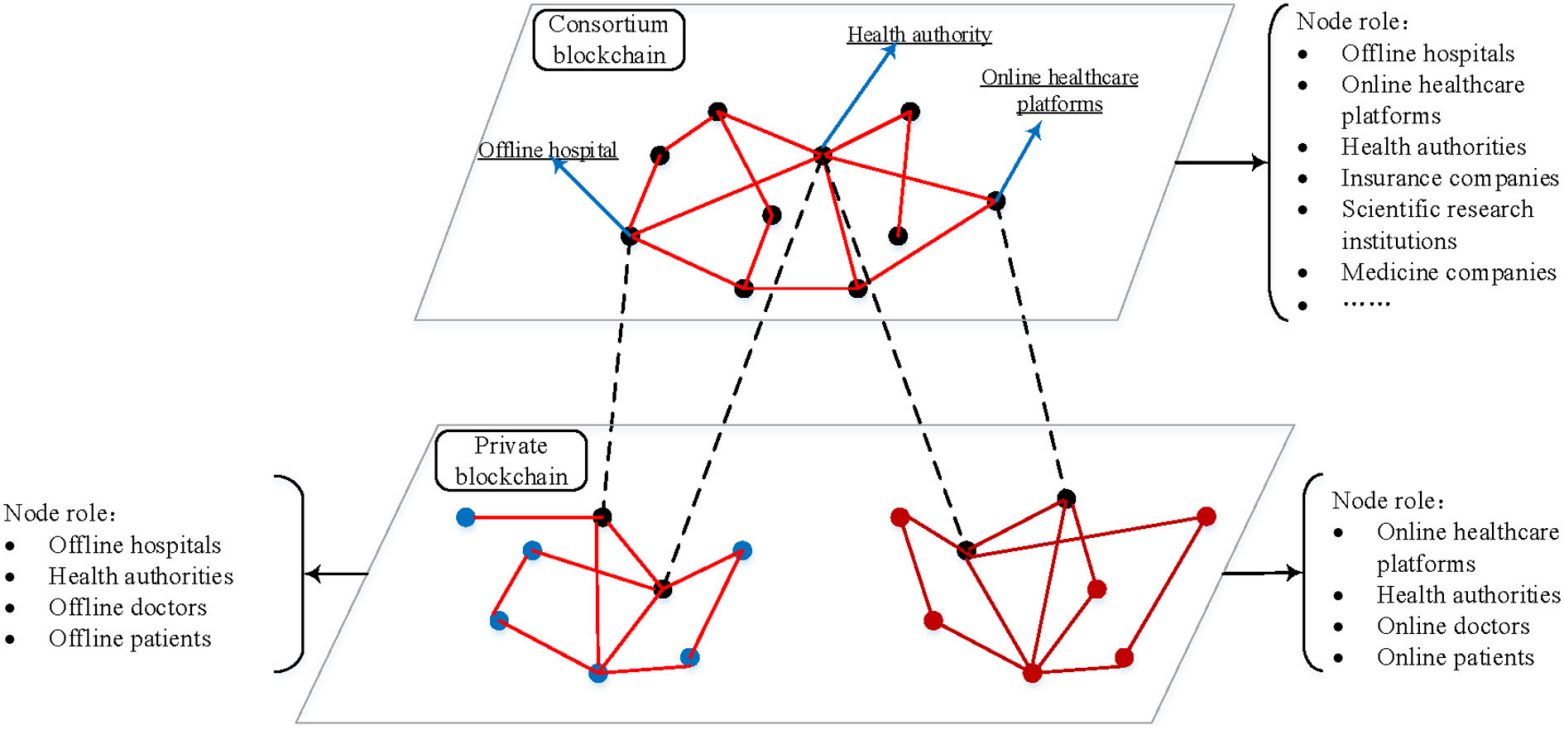
The node network of the MBDS system.

Figure 2 shows the nodes network diagram of the hospitals' private blockchain and consortium blockchain in the MBDS system. The network of the MBDS system is divided into two layers.

(1) The bottom layer is the private blockchain (PBC). It stores the HMI generated within the hospital or on the Internet medicine platform. The original HMI data is generated when the patient visits are stored in the cloud storage server bundled with the blockchain, while the blockchain itself stores the information returned by the cloud storage server. The returned information includes data generation timestamp, transaction type, transaction content, doctor ID, patient ID, encrypted HMI keyword, encrypted HMI storage address, HMI hash value, etc. Executing the preset smart contracts on the private chain can realize the transaction activities between doctors and patients in the hospital. Participants are hospitals or Internet medicine platforms, doctors, patients, and health authorities at all administrative levels.

(2) The upper layer is the consortium blockchain. It stores the information summary of the data that are stored on the underlying hospital private blockchain (e.g., patient ID, encrypted HMI keyword, HMI hash value, HMI stored blockchain ID, Block ID, transaction slip ID, etc.), to facilitate the retrieval of required data by participating node institutions on the consortium blockchain. Executing the preset smart contracts on the alliance chain can realize the transaction activities between participating institutions. Participants are offline hospitals, Internet medicine platforms, health authorities at all administrative levels, medical insurance companies, scientific research institutions, medicine companies, etc.

Figure 2 shows that the private blockchain is divided into two types: the private blockchain of offline hospitals and the private blockchain of the Internet medicine platform. A private blockchain of the offline hospital is only for one hospital, which is used to store and share the HMI of the patient in the offline hospital. The participants are the offline hospital, offline doctors, patients, and health authorities. Besides, a private chain of the Internet medicine platform is only for a platform (e.g., haodf.com), which is used to store and share patients' online HMI generated at this platform. The participants are Internet medicine platforms, online doctors, patients, and health authorities. In both kinds of a private blockchain, the hospital (or Internet medicine platform) and the health authorities at all administrative levels serve as the authority centers and proxies and use a complete client to store a full set of data copies of the located blockchain. Participating doctors can choose to use either a complete client or a lightweight client, and patients can use a lightweight client or an online client. The consortium blockchain between institutions includes numerous hospitals and Internet medicine platforms. This consortium blockchain is connected to the private blockchain that each hospital located through each hospital node and health authority node. In the consortium blockchain, multiple participating institutions are selected as authority centers and proxies, and a complete client is used to store a full set of data copies of the consortium blockchain. In the MBDS system, two types of nodes, the hospital (Internet medicine platform) and the health authorities, play a bridge role in linking the private blockchain to the consortium blockchain. Through these two types of nodes, the communication between the bottom private blockchain and the upper consortium blockchain can be realized. Further, the intercommunication between blockchains realizes the cross-chain combined application of the blockchain.

In particular, in the private blockchain of many different hospitals, there are nodes of the national health authorities and the provincial and municipal health authorities of the region to which the hospitals belong. The health authorities are the common node in the private blockchain of different hospitals, which have the full set of data copies of the hospitals' private blockchains. Thus, other institutions on the consortium blockchain with the authorization of the data owner can access the patient's medical data in other institutions through the health authorities.

By combining multiple hospitals' private blockchains and institutional consortium blockchains, a cross-chain system is established to realize distributed storage and efficient sharing of medical data, while avoiding the disadvantages of large data load, low efficiency, and high energy consumption caused by too many participating nodes on a single blockchain. At the same time, on the premise of ensuring that data stored on the private blockchain can be accessed by other institutions, each hospital's private blockchain can manage its data more efficiently and actively.

Table 1 described the notations used in this study.

**Table 1:** Notation and description.

**Notations**	**Description**	**Notations**	**Description**
*W*	The set of keywords.	$T_{w^{{}^{\prime }}}$	The trapdoor of the searched keyword.
*w*	Keyword.	(*PK*_*i*_, *SK*_*i*_)	The public and private key pair of node *i*.
*p*	Patient.	*address*	The address storing HMI.
*d*	Doctor.	*ID*_*h*_	Hospital's private blockchain ID.
*h*	Hospital.	*ID*_*b*_	Block ID.
*e*	Internet medicine platform.	*ID*_*t*_	Transaction slip ID.
*t*	Timestamp.	*H*()	Hash algorithm.
*Type*	The transaction type.	*SIG*()	Digital signature.
*Text*	The Transaction content.		

### Data structure and storage

The data storage medium of the MBDS system consists of three parts: cloud storage server, block, and transaction slip. The original HMI is encrypted and stored in the cloud storage server bundled with the blockchain. At the same time, the returned information, such as doctor ID, patient ID, encrypted HMI keyword, encrypted HMI storage address, HMI hash value, etc., will be stored in the transaction slips in the block body on the blockchain. Blockchain is composed of blocks that record the previous block ID and hash value, and the body part of each block stores several transactions slip using the Merkle tree structure. These transaction slips are the medium for storing the blockchain data, which are used to store the information records returned from the cloud storage server.

**Figure 3:**
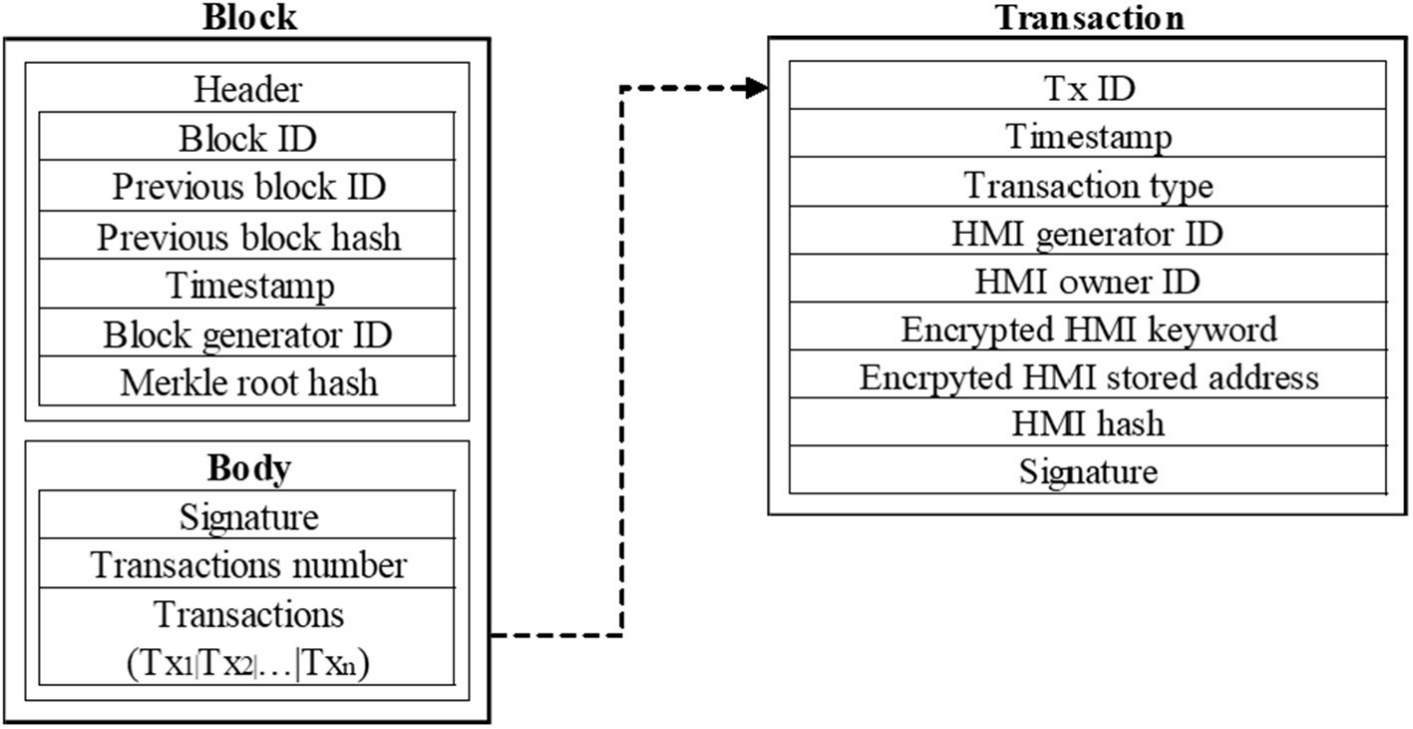
Structure of a block in the hospital's private blockchain.

In the MBDS system, the functions of the private blockchain and consortium blockchain are different, and the information stored on the blockchain is different. Therefore, the data storage structure of the blocks in the two types of blockchains is also different. Figure 3 shows the data storage structure of a block in the bottom private blockchain of the hospital. The block header contains the current block ID, the ID and hash of the previous block, the timestamp of creating the block, the user ID of the creator of the current block, and the root hash value of the Merkle tree. The block body contains the digital signature, transaction number, and transaction slip. Among them, the digital signature in the block body is to ensure that the content of the block is not tampered with and to ensure that the block creator cannot deny it after generating a malicious block. Transaction slips are stored in a Merkle tree structure, and each transaction slip is stored in a leaf node. When the cloud storage server stores the HMI, it will add the data generation timestamp, transaction type, doctor ID, patient ID, and HMI hash value into the data. After storing the data, this information will be returned to the blockchain and stored in the transaction slip. Therefore, in the design of the transaction slip storage structure, the storage data includes transaction slip ID, data generation timestamp, transaction type, transaction content, doctor ID, patient ID, encrypted HMI keyword, encrypted HMI storage address, HMI hash value, and digital signature. The digital signature is to ensure that the transaction content is not tampered with and to ensure that the creator cannot deny it after generating a malicious transaction slip.

It is noted that in the MBDS system, a transaction slip has an ID, which ID only points to the HMI stored at the HMI storage address in the transaction slip. Separating the original HMI from the stored data on the blockchain, can reduce the data capacity on the blockchain, facilitate synchronization and backup, and improve the operational efficiency of the blockchain. Blocks and transaction slips are physically stored in the database, and logically stored in the form of a blockchain, to realize the unmodifiable, traceable, and efficient sharing of data.

**Figure 4:**
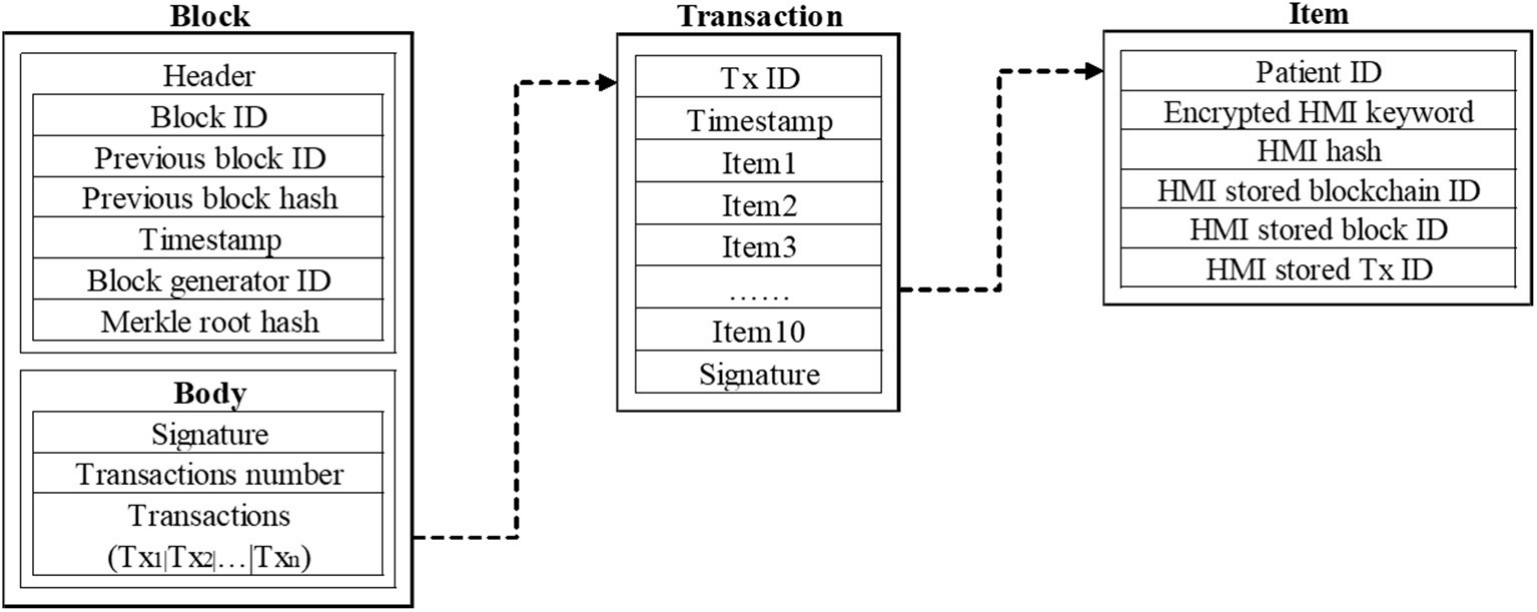
Structure of a block in the consortium blockchain.

Figure 4 shows the data storage structure of a block in the upper consortium blockchain between institutions. The block is used to store the information summary of the data stored in the private blockchain of the hospital, to facilitate the retrieval of the required data by each institutional node on the consortium blockchain. The hospital node in the consortium blockchain will regularly submit the information summary of their private blockchain to the consortium blockchain and store it in the block. The header of the block in the consortium blockchain is the same as that in the private blockchain. The difference is that the transaction slip in the block of the consortium blockchain contains a transaction slip ID, transaction slip generation timestamp, 10 items, and a digital signature. Among them, the item is used to store the information summary of the HMI stored on the private blockchain submitted by the hospital. The item contains the patient ID, encrypted HMI keyword, HMI hash value, HMI stored blockchain ID, block ID, and transaction slip ID. When a hospital needs to know the electronic medical records of a patient in other hospitals, by obtaining authorization from the patient, as well as the patient ID and the trapdoor $T_{w^{{}^{\prime }}}$ of the searched keywords, the hospital can retrieve the required information in the consortium blockchain, and find the storage information, such as blockchain ID, block ID, and transaction slip ID, then the health authorities, as the proxy, will obtain the patient's HMI and share with the prior hospital.

### Consensus mechanism

Due to the different participating groups and functions, the consortium blockchain and private blockchain in the MBDS system adopt different consensus mechanisms. The consortium blockchain uses the DPoS algorithm as the consensus algorithm, and the hospital private chain uses the PBFT algorithm as the consensus algorithm. Each node in the institutional alliance chain is relatively stable with few changes. Using the DPoS consensus mechanism can give full play to the role of each representative node. The DPoS consensus mechanism has many advantages, but when starting to use the DPoS consensus mechanism, the initial method of selecting the representative node is through the election of node voting. This method may have problems when applied to the medical blockchain, namely: the initially selected representative node may not have enough influence and the ability to provide the full range of services such as full copy data storage and query. Therefore, the MBDS system has improved the initial selection method of the representative node of DPoS. According to the current rating of medical institutions in China, the deployment scale of information centers, and enterprise strength, participating institutions are scored from multiple dimensions to obtain credit scores. Then, we select the top 101 institutions with high credit scores who are willing to be representatives as the representative node (RPN) and select the top 102–121 as the audit node (ATN) (15). RPN is responsible for creating blocks in turn to save the data submitted to the blockchain by each node, and ATN, in turn, verifies the authenticity and validity of each created block.

The ranking of institutions is based on credit scores, which indicates the comprehensive strength of the institutions. The credit score is bound to the institution's identity ID. Providing services for the operation of the blockchain and data sharing can increase own credit score. Actions that disrupt the stable operation of the blockchain, such as RPN creating an invalid or incorrect block, or ATN verification errors, will deduct the institution's credit score. When the credit score is lower than the set threshold, the institution will be kicked out of the RPN group and replaced by ATN in turn. Nodes in the ATN group will be replaced by nodes with high subsequent credit scores. By introducing the credit score system to assess the performance of RPN and ATN, it can fully mobilize the initiative of participating institutions to participate in the evaluation, and provide a reference for the evaluation of the ability of representative institutions. In addition, in the process of nodes jointly maintaining the operation of the blockchain, the credit score system evaluates the performance of each node in a quantitative way, which improves the rationality of group decision-making.

The private blockchain of the hospital in the MBDS system adopts the PBFT algorithm as the consensus mechanism. This algorithm requires the minimum number of participating nodes to be 4, which effectively solves the shortcomings of the DPoS algorithm in that the number of participating nodes in the blockchain system is always more than 101. It can meet the requirements of large and flexible nodes in the hospital's private blockchain (8). In addition, compared with POW and POS algorithms, PBFT does not need to spend computing power to solve mathematical problems, saving energy and higher efficiency.

### Process of MBDS system

**Figure 5:**
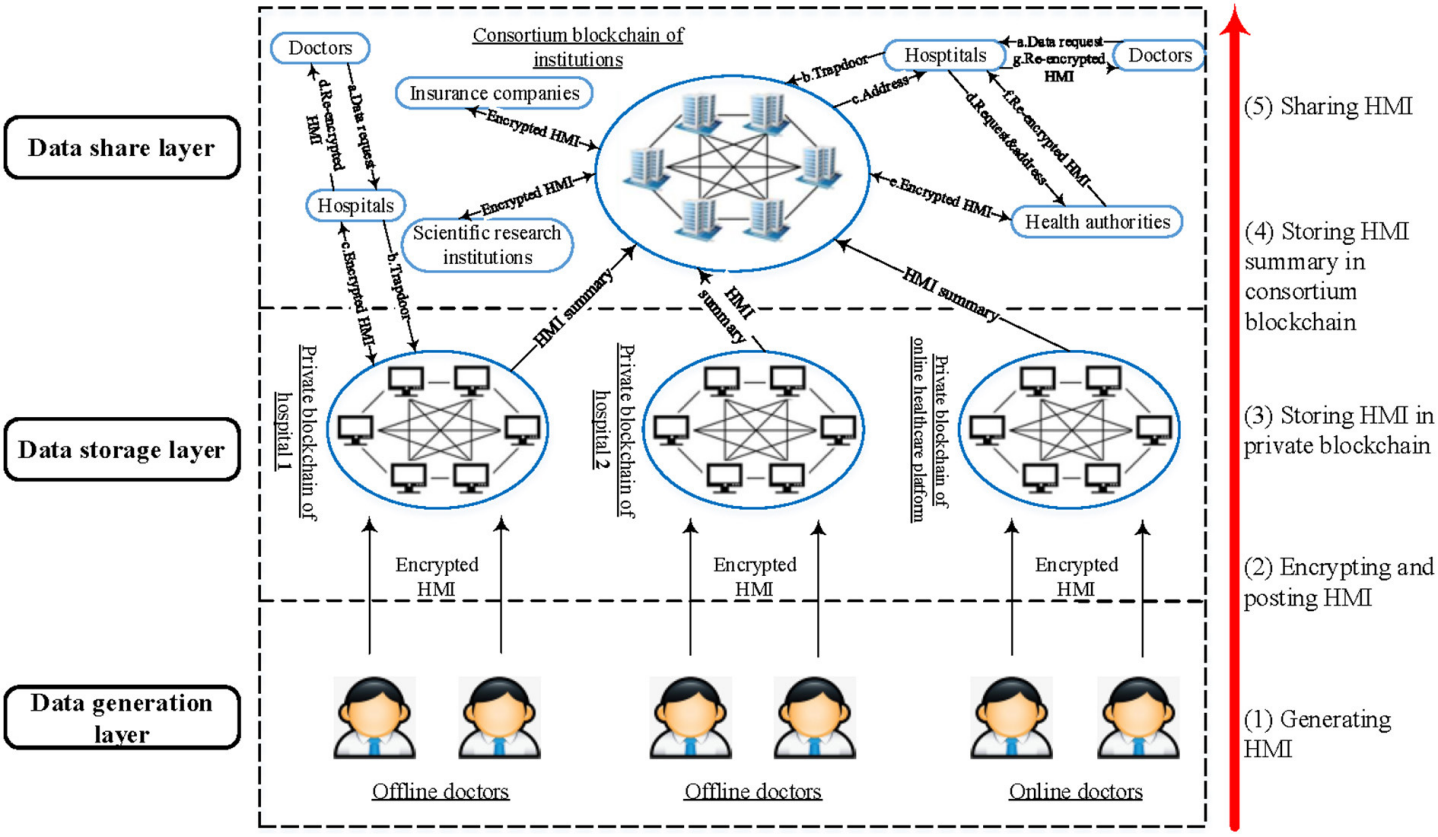
The protocol in the MBDS system.

Medical data sharing is an important step to realizing an intelligent medical system and improving medical service quality (31), which can also help patients become active participants (44, 45), improve the quality of medical services (46), provide patients and doctors with better recommendations and diagnosis and treatment recommendations (47). In this paper, MBDS systems which are designed based on blockchain technology can help hospitals, patients, and service providers quickly and securely authenticate permissions and achieve efficient data access and sharing. Based on blockchain technology, patient medical data can be obtained quickly and accurately, and better medical services can be provided to patients, which is conducive to building an equal and credible medical ecological environment. When a patient visits a doctor, medical data in the MBDS system goes through four steps from generation to sharing and spans the data generation layer, data storage layer, and data share layer, as shown in Figure 5.

#### System initialization

In the initial operation of the MBDS system, the participants (hospitals, Internet medicine platforms, doctors, health authorities, medical insurance companies, etc.) need to authenticate to the Key Management Center (KMC) and request the generation of public and private key pair (*PK*_*i*_, *SK*_*i*_). A participating node can only apply for one public and private key pair. The real identity of the node is bound to the public and private key pair, and the KMC encrypts and protects the identity information of the node. In this system, the public key *PK*_*i*_ of a user is used as the user's identity ID. When a patient visits a doctor for the first time, the patient *p* will go to the hospital *h* and complete the identity verification with the assistance of the hospital *h* and at the same time authenticate to the KMC to obtain his public and private key pair (*PK*_*p*_, *SK*_*p*_). This public and private key pair will be the patient's unique identity ID information in the MBDS system, which can be used by the patient later when visiting other hospitals or Internet medicine platforms.

#### HMI data generation and release

The patient *p* visits the doctor *d* in the hospital *h*, doctor *d* will write the electronic medical records after medical examinations and treatment. All the information is aggregated to form the patient's HMI. The doctor extracts keywords *w* according to HMI to describe the patient's disease or symptoms. It is noted that the keywords need to comply with unified standards (such as the FHIR standard, a standard used to specify data formats and elements for the exchange of electronic medical records, to promote interoperation between healthcare systems). After generating the HMI *M* and the keywords *w*, firstly, the doctor *d* calculates the hash value *H*(*M*) and then uses the patient's public key *PK*_*p*_ to encrypt the HMI and keywords to get the ciphertext *C*_*M*_ and *C*_*w*_. After that, the doctor uses his private key *SK*_*d*_ to digitally sign the uploaded data. Finally, doctor *d* uploads the encrypted data and digital signature to the private blockchain *ID*_*h*_ of the belonged hospital *h*. The specific process is shown as Algorithm 1 in Table 2. The cloud storage server bundled with the blockchain stores the data and the blockchain executes the preset smart contract and notifies the patient *p* to confirm it after verification.

**Table 2:** Pseudocode of encryting data.

**Algorithm 1**	**Encrypting data**
**Input:**	HMI *M*, keywords *w*, patient's public key *PK*_*p*_, doctor's public key *PK*_*d*_, doctor's private key *SK*_*d*_, timestamp *t*, transaction type *Type*, transaction content *Text*.
**Output:**	Ciphertext *C*_*M*_, *C*_*w*_, digital signature *Sig*.
1:	*C*_*M*_ = *Enc*(*PK*_*p*_, *M*);
2:	*C*_*w*_ = *Enc*(*PK*_*p*_, *w*);
3:	*Sig* = *SIG*(*SK*_*d*_, *t*|*Type*|*Text*|*PK*_*d*_|*PK*_*p*_|*H*(*M*)|*C*_*M*_|*C*_*w*_);
4:	**Return** *C*_*M*_, *C*_*w*_, *Sig*.

#### Data storage

After the doctor generated and uploaded the HMI to the private blockchain, the patient will receive a notification from the client. The patient first verifies the doctor's signature and uses the private key *SK*_*p*_ to decrypt the received ciphertext to obtain the plaintext *M* and *w*. The specific process is shown as Algorithm 2 in Table 3. After reviewing the HMI and related information, the patient will use the private key *SK*_*p*_ to encrypt and sign the data, the process is similar to Algorithm 1. During this process, the patient does not have permission to write or modify the data. So far, the data verification process of the patient has been completed. The private blockchain system stores the information confirmed by the patient according to the preset data structure, and returns the timestamp, transaction type, transaction content, doctor ID, patient ID, encrypted HMI keywords (*Enc*(*SK*_*p*_, *w*)), encrypted HMI storage address (*Enc*(*SK*_*p*_, *address*)) and HMI hash value. The returned information is stored in the transaction slip automatically, and at the same time, the patient digitally signs the transaction slip. When 128 transaction slips are accumulated in the private blockchain or 10 min later, the primary node determined by the PBFT consensus mechanism will create a block to store the accumulated transaction slips. The data storage structure of a block in the hospital's private blockchain is shown in Figure 3.

**Table 3:** Pseudocode of decrypting data.

**Algorithm 2**	**Decrypting data**
**Input:**	Ciphertext *C*_*M*_, *C*_*w*_, patient's private key *SK*_*p*_.
**Output:**	HMI *M*, keywords *w*.
1:	*M* = *Dec*(*SK*_*p*_, *C*_*M*_);
2:	*w* = *Dec*(*SK*_*p*_, *C*_*w*_);
3:	**Return** *M*, *w*.

Each hospital extracts the information summary from the data stored in each own private blockchain and submits it to the consortium chain for storage. When 10 new blocks are created on the private blockchain or 10 min later, the hospital on the private blockchain will extract the data summary from the stored transaction slips and upload them to the consortium blockchain. The data storage structure is shown in Figure 4. A block stores several transactions slip, a transaction slip contains 10 items, and an item stores the information summary of a transaction slip on the private chain (patient ID, encrypted HMI keywords, HMI hash value, HMI stored blockchain ID, block ID, and transaction slip ID). When the number of transaction slips accumulated in the consortium blockchain reaches 128 or 10 min later, the RPN determined by the DPoS consensus mechanism will create a block on the consortium blockchain, and ATN will be responsible for verifying the block.

#### Data sharing

The patient has full control over the access rights of his HMI, the patient can control data transmission and sharing by controlling the access rights. To ensure secure sharing of HMI and improve data access efficiency, patients can preset access permissions in the smart contract of blockchain, such as access permissions, access actions (such as read, write or copy), duration, etc. Once the access conditions are met, the smart contract will automatically trigger and perform corresponding operations to ensure the validity and fairness of data sharing. For nodes that are not in the set of nodes with preset access permissions, an application needs to be submitted to the patient, and the applicant can obtain the data access permission after the patient's confirmation.

When a patient *p* goes to a hospital *h*, a doctor *d* in this hospital thinks that it is necessary to know the patient's historical medical records, then the doctor can apply for access authorization from the patient and review the patient's relevant medical records. In the MBDS system, we can divide the data retrieval and share it into three scenarios. The first scenario is that the patient only visits the doctor in one hospital and has never been to other hospitals; the second is that the patient has treatment experience in multiple hospitals; the third is that the patient has treatment experience in many offline hospitals and Internet medicine platforms. There are some differences in data sharing among the three scenarios, Algorithm 3 in Table 4 shows the core steps in sharing HMI in the MBDS system.

**Table 4:** Pseudocode of sharing data.

**Algorithm 3**	**Sharing data**
**Input:**	Patient's public key *PK*_*p*_, patient's private key *SK*_*p*_, searched keywords *w*′, doctor's public key *PK*_*d*_, doctor's private key *SK*_*d*_.
**Output:**	HMI *M*.
1:	Patient *p* generates the trapdoor of searched keywords *w*′ and sends to doctor *d*: $T_{w^{{}^{\prime }}}=Trapdoor\left(SK_{p},\;w^{{}^{\prime }}\right)$*;*
2:	Doctor *d* sends *PK*_*p*_ and $T_{w^{{}^{\prime }}}$ to the proxy; then, the proxy checks whether there are records in the blockchain that the encrypted HMI keywords corresponding to *PK*_*p*_ match the trapdoor $T_{w^{{}^{\prime }}}$, if any, the proxy will obtain the encrypted HMI storage addresses stored in the transaction slips;
3:	Patient *p* decrypts the encrypted HMI storage addresses, then encrypts the addresses using the proxy's public key, and at the same time generates the re-encryption key *RK*_*p*→*d*_ for the proxy;
4:	The proxy accesses the encrypted HMI according to the received HMI storage addresses and re-encrypts the encrypted HMI to obtain re-encrypted HMI *C*_*M*←*P*_*K*__*d*__ = *ReEnc*(*C*_*M*_, *RK*_*p*→*d*_);
5:	Doctor *d* decrypts the re-encrypted HMI sent by the proxy to obtain the plaintext: _*M*_*i*_ = *Dec*(*SK*_*d*_, *CM*←*PK*_*d*__);
6:	Combine the multiple decrypted HMI to get integrated medical records: *M* = *Combine*(_*M*_1_, *M*_2_, …, *Mn*_);
7:	**Return** *M*.

In addition, as for the possible security vulnerabilities of the MBDS system, the MBDS system has established a security vulnerability identification mechanism. In terms of infrastructure components and facilities, the key points who provide infrastructure components and facilities must provide security protection for facilities and offline databases. In terms of identity theft, the system monitors the access times and IP addresses of the different types of users. If there is abnormal access, the node is temporarily disabled until the user identification is confirmed.

## The process of a patient using the MBDS system

By integrally managing and distributing storing the healthcare information in the different offline hospitals and Internet medicine platforms, the MBDS system designed in this study aims to resolve the information silos and information fragmentation problems caused by the traditional centralized way of storing and sharing healthcare information, to integrate the online medical services provided on Internet medicine platforms into the current official healthcare system. In this way, we can achieve efficient and complete medical data sharing between various online and offline hospitals, and finally, provide a feasible solution to build an equal and credible medical ecological environment in the whole society. In an equal and credible medical ecological environment, all parties involved in the medical industry are equal and mutually trusted, the status between doctors and patients is equal, and information is transparently shared. Information silos, crises of confidence, and moral hazards will all be addressed to a certain extent.

In the established, equal, and credible medical ecological environment, through the MBDS system, the integration of offline medical care and online medical care can promote the seamless connection of online and offline medical treatment services, which helps to achieve the goal of hybrid medical treatment in both online and offline channels. In this way, both online and offline doctors can grasp the patient's disease information and historical electronic medical records, so there is no need to conduct secondary medical examinations and repetitive doctor-patient conversations. In addition, by referring to a patient's complete electronic medical records, doctors can conduct a unified analysis of the physical symptoms and treatment plans of patients in different periods and find potential or easily overlooked causes of disease, to facilitate a more careful and comprehensive diagnosis and treatment for patients.

To better understand, this section takes the complete process of a patient's online and offline medical treatment as an example to illustrate how to implement online and offline hybrid medical applications based on the MBDS system in an equal and credible medical ecological environment.

- Step 1, visit doctors on Internet medicine platforms. When a patient feels unwell, he will look up relevant information on the Internet medicine platforms (such as haodf.com), and infer the diseases he may have based on their symptoms. The patient will find appropriate an online doctor on the Internet medicine platform to seek online medical services. The patient can communicate with the doctor through text messages, voice messages, or phone calls. The doctor can make a preliminary diagnosis of the disease that the patient may have based on the patient's descriptions. If the doctor thinks that the patient's symptoms are normal or will heal and improve naturally, there is no need to worry about it too much. Afterward, the online doctor will generate HMI for the patient and submit the record to the private blockchain of the using Internet medicine platform. Then the patient will check and confirm the generated HMI. The encrypted HMI is stored in the private blockchain of the Internet medicine platform and the bundled cloud storage server. Hereto, the doctor and patient complete the online medical consultation service.
- Step 2, visit doctors in offline hospitals. If the above patient is not satisfied with the diagnosis result of the online doctor, or the doctor thinks that the patient needs to go to an offline hospital for necessary medical examinations and diagnosis, then after the first step, the patient will go to the offline hospital to find an appropriate doctor to seek offline medical service. The doctor in the offline hospitals has a preliminary understanding of the patients, and then obtains the patient's historical electronic medical record on Internet medicine platforms in the MBDS system after obtaining the patient's authorization. Then, the offline doctor will arrange medical examinations and treatment based on the medical records generated by online doctors and his medical knowledge and experience. Afterward, the offline doctor will generate HMI for the patient and submit it to the blockchain, the patient will check and confirm the generated HMI. The encrypted HMI is stored in the private blockchain of the offline hospital and the bundled cloud storage server. Hereto, the doctor and patient complete the offline medical service.
- Step 3, visit a different offline doctor or go to another offline hospital. If the above patient is not satisfied with the doctor he visited in the second step, he can choose to seek medical service from a different doctor or expert in the same hospital, or go to another hospital for treatment. When a patient goes to another doctor or hospital, the subsequent doctor can access the patient's historical electronic medical records through the data sharing operation in the MBDS system for further diagnosis and treatment. Information such as diagnosis results and treatment plans are also stored in the private chain of the belonging hospital. Hereto, the doctor and patient complete the online medical referral service.
- Step 4, online follow-up service on Internet medicine platforms. When the patient completes the diagnosis and treatment of the doctors in step 2 or Step 3, he can go to the Internet medicine platforms and seek online follow-up services from previously visited offline doctors. Online doctors can access the patient's historical electronic medical records and inquire about his recent physical condition, follow up on the patient's physical recovery and disease treatment, realizing closed-loop control. Online doctors will generate HMI for the patient based on the information during follow-up service and submit the HMI to the private blockchain. Hereto, doctors and patients complete the online follow-up service.

Through the above four steps, the MBDS system can realize patients' treatment between online hospitals and offline hospitals, and can also realize the referral between different offline hospitals or different online hospitals, providing technical support for the realization of online and offline hybrid medical care. Through the MBDS system, the medical resources and HMI of offline medical institutions (such as community hospitals, specialist hospitals, general hospitals, rehabilitation hospitals, scientific research institutions, etc.) and Internet medicine platforms can be systematically integrated to achieve efficient data sharing and incorporate the online medical service into the current healthcare system, to realize online and offline hybrid medical care. The designed system and proposed solution can solve the problems of medical information interconnection and resource sharing, improve the utilization efficiency of medical resources (including doctors and medical equipment), improve the efficiency and effectiveness of disease diagnosis, ease doctor-patient conflict, and improve the doctor-patient relationship.

## Conclusions

This study used blockchain technology to store encrypted health and medical information in distributed storage mode and systematically integrated the health and medical information of patients in offline medical institutions and Internet medicine platforms, to achieve equality, credibility, and data sharing among participating nodes. To improve the data security on the blockchain, reduce the computational pressure and avoid the situation of too many nodes in the blockchain, this study built the private blockchains used for the hospitals and a consortium blockchain used between institutions, respectively, then combined the consortium blockchain and private blockchain to design the MBDS system. In terms of data storage, the system used the combination of blockchain and cloud storage to store medical data, which can meet the large-capacity demand of medical data while ensuring the safe storage of data. Besides, PEKS technology was used to achieve data retrieval on the premise of protecting patient privacy. In terms of data sharing, the MBDS system adopted PRE technology to facilitate data sharing and enhance data reliability and security while reducing the users' burden. By using the MBDS system, patients can achieve a seamless connection between Internet medicine platforms and offline hospitals, realize the efficient sharing of medical information, integrate online medical services into the current official healthcare system, and promote online and offline hybrid medical care. The realization of this will improve the utilization efficiency of medical resources, solve the problems of “difficult in seeing a doctor” and “inconvenient in seeing a doctor,” and finally realize the purpose of alleviating doctor-patient contradiction and improving the doctor-patient relationship.

Currently, the medical industry is still trying various ways for storing and sharing health data. However, the existing medical data sharing system cannot achieve universal cross-regional and cross-institutional data sharing and application, and patients have no control over their medical record data. Most importantly, the current data-sharing system only realizes the sharing application of medical data, which cannot guarantee the accuracy of data and cannot provide endorsement for data. Therefore, the credibility and effectiveness of patient medical data in the process of cross-organization use are greatly reduced. The medical industry in the whole society cannot form a credible medical ecological environment. In contrast, the MBDS system designed in this study is based on the features of blockchain technology such as tamper-proof, traceability, decentralization, and joint participation, which realized the safe storage of medical data. Based on digital signatures, the doctor's endorsement of the patient's medical data ensures the accuracy and credibility of the data. In general, the system can achieve the goals of medical data security storage, tamper-proof, traceability, data sharing, and permission control, user privacy protection, information security retrieval, etc., which improves the shortcomings of the existing medical data sharing system and can better help build an equal and reliable medical ecological environment.

This study has some theoretical and practical implications. In terms of theoretical implications, firstly, this study put forward for the first time the concept of using medical blockchain to build an equal and credible medical ecological environment, which provided a new idea for the future development of the medical and health field. Secondly, from the perspective of cross-chain and double-chain, this research combined private chains and alliance chains to build a medical blockchain double-chain system. And at the same time, the designed system adopted different consensus algorithms in different types of blockchain. This study provided a new way for data storage and sharing in the medical industry, and provided a new idea for the design of a medical blockchain system. In terms of practical implications, the MBDS system constructed in this study and the proposed online and offline hybrid medical treatment mechanism incorporated online medical services into the current healthcare system and provided new solutions and practical guidance for the future development of collaborative medical care.

This study has several limitations, in the future, we will do further research in the following scope. Firstly, in terms of data retrieval, the use of PEKS technology can only realize the encrypted search of a single keyword, and the system through multiple searches to achieve multiple keyword retrieval. In the future, conjunctive keyword searchable encryption technology can be considered to reduce the number of searching times and improve retrieval efficiency. Secondly, in terms of data storage, the MBDS system used a combination of blockchain and cloud storage servers to store large-capacity data, but the storage of original data relies on cloud storage servers. Therefore, the choice of cloud storage providers needs to be extra cautious, and it is necessary to introduce multiple suppliers. The distributed storage of data will be distributed on multiple cloud storage servers to ensure data security.

## Data availability statement

The original contributions presented in the study are included in the article/supplementary material, further inquiries can be directed to the corresponding author.

## Author contributions

CL, JL, ZW, and JH: conceptualization. CL: methodology. CL and ZW: formal analysis. CL, JL, and GQ: writing- original draft. CL, JL, GQ, ZW, and JH: writing-review and editing. CL and JH: funding acquisition. GQ: literature. All authors contributed to the article and approved the submitted version. All authors listed have made a substantial, direct, and intellectual contribution to the work and approved it for publication.

## Funding

This work was supported by the Major Program of the National Fund of Philosophy and Social Science of China (grant no. 18ZDA088), supported by research project of Shanghai University of Sport (grant no. 2022XJ024), and support by Shanghai Universities Young Teacher Training Funding Program.

## Conflict of interest

The authors declare that the research was conducted in the absence of any commercial or financial relationships that could be construed as a potential conflict of interest.

## Publisher's note

All claims expressed in this article are solely those of the authors and do not necessarily represent those of their affiliated organizations, or those of the publisher, the editors and the reviewers. Any product that may be evaluated in this article, or claim that may be made by its manufacturer, is not guaranteed or endorsed by the publisher.

## Abbreviations

ATN: audit node
DPoS: delegated proof of stake
EHRs: electronic health records
HMI: health and medical information
KMC: key management center
MBDS: medical blockchain double chain system
PoS: proof of stake
PoW: proof of work
PoI: proof of information
PBFT: practical byzantine fault tolerance algorithm
RPN: representative node.
